# Identification of a molecular subtyping system associated with the prognosis of Asian hepatocellular carcinoma patients receiving liver resection

**DOI:** 10.1038/s41598-019-43548-1

**Published:** 2019-05-08

**Authors:** Xiaohua Ma, Jingxian Gu, Kun Wang, Xing Zhang, Juan Bai, Jingyao Zhang, Chang Liu, Qiang Qiu, Kai Qu

**Affiliations:** 1 Department of Hepatobiliary Surgery, The First Affiliated Hospital of Xi’an Jiaotong University, Xi’an, 710061 Shaanxi China; 2 Center for Ecological and Environmental Sciences, Key Laboratory for Space Bioscience and Biotechnology, Northwestern Polytechnical University, Xi’an, 710072 China; 3 Department of Immunology, Shaanxi University of Chinese Medicine, Xianyang, Shaanxi 712046 People’s Republic of China

**Keywords:** Prognostic markers, Liver cancer

## Abstract

Hepatocellular carcinoma (HCC) remains a severe health issue worldwide, especially in Asia. To date, molecular classifications proposed for the overall survival (OS) or recurrence-free survival (RFS) prediction of Asian HCC patients after hepatectomy are quite few and limited in clinical practice. Here, we established a molecular subtyping system for Asian HCC to facilitate prognosis evaluation. Firstly, differentially expressed genes (DEGs) (FDR [false discovery rate] <0.05) between different types of liver cancer and non-tumor tissue were screened. Among the DEGs solely between HCC and non-tumor samples, 185 genes simultaneously significantly associated with the OS and RFS were identified as HCC-characteristic genes. The molecular subtypes were developed based on the expression profiles of the 185 genes in the training dataset (TCGA [The Cancer Genome Atlas] dataset) using non-negative matrix factorization (NMF) clustering method. Patients were then classified into Subtype1 and Subtype2 groups denoting unfavorable and favorable clinical outcome respectively. The robustness and effectiveness of the molecular subtype was confirmed in another independent dataset (GSE14520) by the same clustering approach and Kaplan-Meier analyses. Moreover, functional prediction analysis revealed that the identified molecular signature was involved in chemotaxis, apoptosis and cell development associated pathways. Besides, the molecular signature was closely related to the clinical characteristics including TNM stage, preoperative alpha-fetoprotein (AFP) level and *TP53* mutation. Furthermore, integration of the molecular subtype and TNM stage was demonstrated to improve risk stratification. Taken together, our molecular subtyping system exhibited great utility and potential in prognosis prediction and therapeutic decision making of Asian HCC patients.

## Introduction

Hepatocellular carcinoma (HCC) is the major type of primary liver cancer (PLC), known as one of the leading causes of cancer-related mortality^1^. HCC is most prevalent in eastern and north-eastern Asia due to the high rate of hepatitis B infection and exposure of aflatoxin B1 in this area^2^. Only China accounts for about half of globally new-arisen and dead cases^3^. Despite large improvement in therapeutic approaches over the decades and patients’ discomfort can be temporarily relieved, the postoperative overall survival (OS) of HCC patients has not prolonged much^4,5^.

To date, many staging systems have been proposed for the risk stratification of HCC. But there might be inconsistency between different stages even for some conventional systems like AJCC TNM stage, Barcelona Clinic Liver Cancer (BCLC) classification, etc.^6^. One of the important reasons for the limited use in clinical practice is that the present systems are all based on the clinical presentations or laboratory tests. None of these staging methods involves any molecular or genetic characteristics. While HCC is an extremely heterogenous disease and the intrinsic heterogeneity might have no direct relations to what patients present.

Extensive efforts have been made in investigating the molecular features of HCC which might partly reveal the underlying tumor biology. Recent evidence showed that molecular classifications based on genetic information are promising methods of subclassifying the heterogenous HCC for accurate survival prediction^7^. However, by far, only a handful of prognostic molecular staging systems have been proposed and all of them are lack of widely external validation^8^. Therefore, in this present study, we aimed to construct a robust and effective molecular subtyping systems for evaluating the prognosis of HCC patients after surgery.

## Results

**Figure 1:**
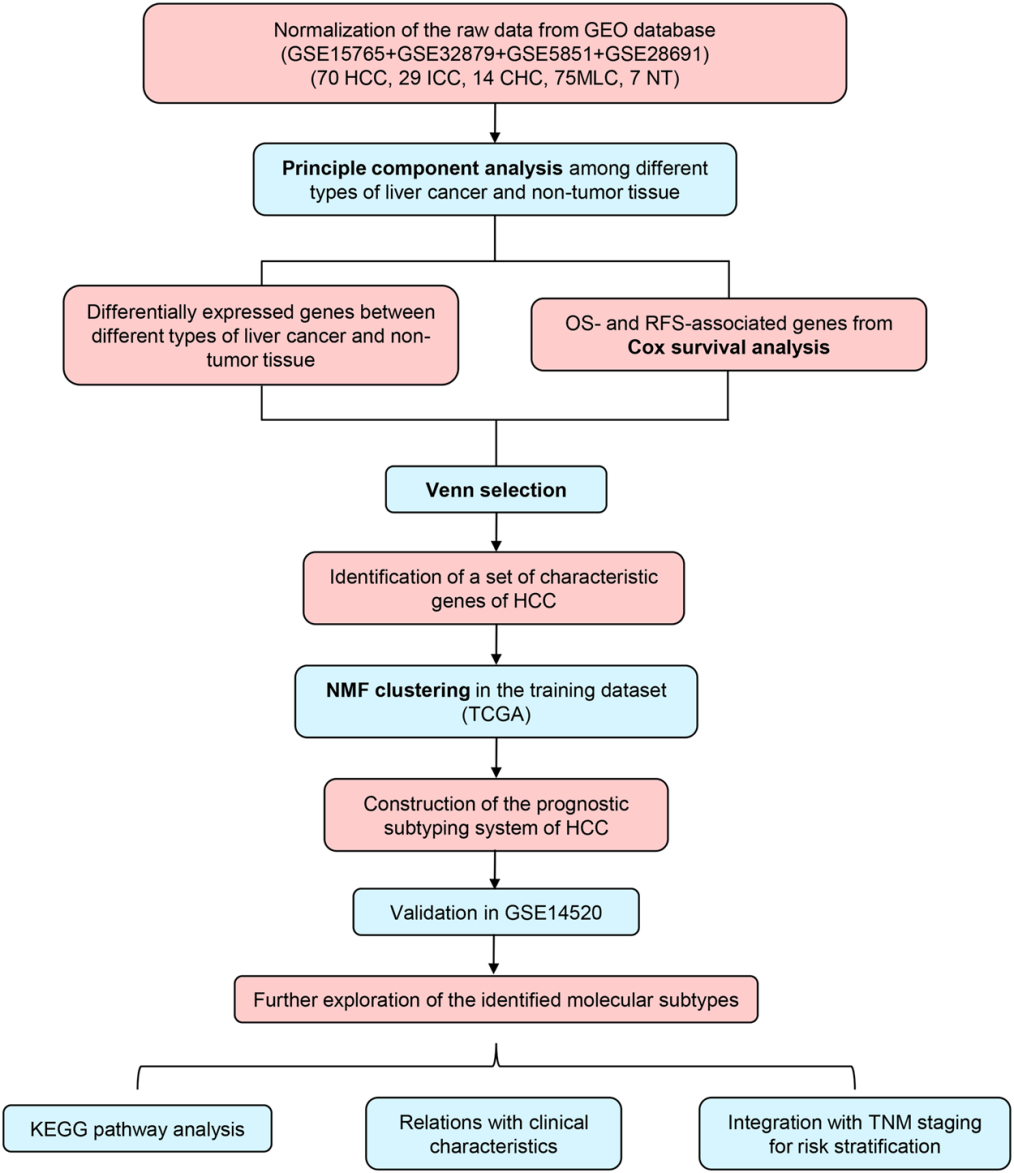
Flow chart of the analytic procedures for this study.

**Figure 2:**
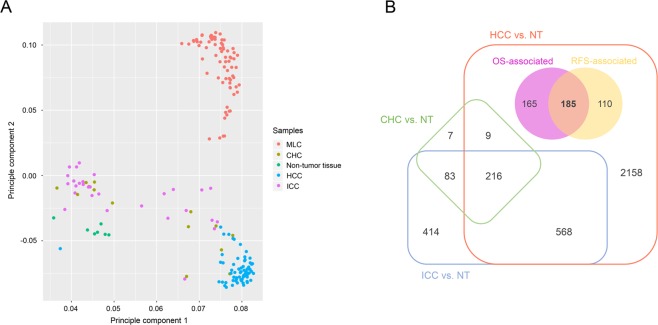
Identification of HCC-characteristic genes in Asian patients who received liver resection. (**A**) Scatter plot of the two-dimension principle component analysis (PCA) among different types of liver cancer and non-tumor liver tissue. (**B**) Venny diagram of the screening of HCC-specific genes. The numbers of the differentially expressed genes (DEGs) (FDR <0.05) between three histological types of primary liver cancer and non-tumor samples were shown in border-radius squares. The two circles in HCC vs. NT region without overlapping the other two cancers denote the prognostic DEGs of HCC solely differentially expressed between HCC and non-tumor tissue.

The study design was presented in Fig. 1. As shown in Fig. 2A, principal component analysis (PCA) of four types of liver cancer and non-tumor samples showed a clear separation between HCC and non-tumor or metastatic liver cancer (MLC). Besides, intrahepatic cholangiocarcinoma (ICC) and combined hepato-cholangiocarcinoma (CHC) were totally detached from MLC as well, indicating there was almost no relationship between primary liver cancer (PLC) and MLC. In case of three histological types of PLC, although a small proportion of ICC or CHC samples were quite close to HCC or even overlapped some HCC samples, HCC was separated from most ICC and CHC samples. Anyway, the PCA results gave implications that the construction of a reliable Asian-HCC molecular subtype system should be on the basis of the specific gene set of Asian HCC.

The numbers of the significantly expressed genes (FDR [false discovery rate] <0.05) between three types of PLC (HCC: n = 70, ICC: n = 29, CHC: n = 14) and non-tumor liver (n = 7) and of the common differentially expressed genes (DEGs) in two or three subtypes were presented in Venn diagram (Fig. 2B). For those genes merely significantly expressed in HCC versus non-tumor samples (n = 2618), the relationships of their expression level and the OS or RFS [recurrence-free survival] of the patients was examined. A total of 185 genes were significantly associated with the OS and RFS of Asian HCC patients after surgery simultaneously (Cox *P* value < 0.05) (Fig. 2B). This set of 185 genes were identified as HCC-characteristic prognostic gene signature for the development of the molecular subtypes of HCC (Table S1).

**Figure 3:**
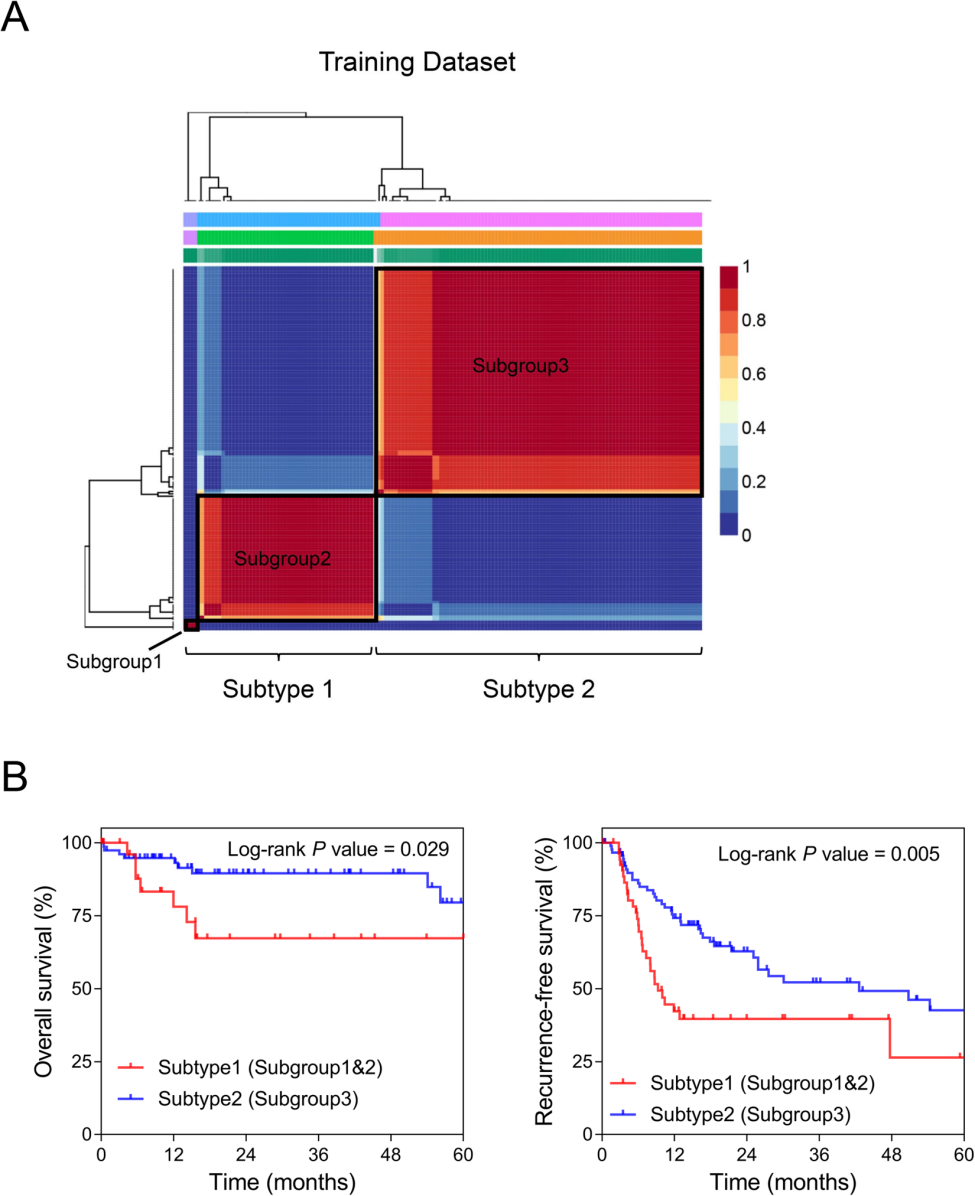
Establishment of the molecular subtypes of Asian HCC. (**A**) The consensus map of the non-negative matrix factorization (NMF) clustering results in the training dataset of 150-TCGA samples. Subgroup1 and Subgroup2 were classified as molecular Subtype1 while subgroup3 was categorized as molecular Subtype2 according to the expression profiles of 185 HCC-characteristic genes. (**B**) The Kaplan-Meier plots comparing the overall survival (OS) (*Left*) and recurrence-free survival (RFS) (*Right*) of molecular Subtype1 and Subtype2 patients. *P* values were calculated by Log-rank tests.

Non-negative matrix factorization (NMF) clustering analysis was applied to the gene set of the Asian HCC from The Cancer Genome Atlas (TCGA) project. As shown in the consensus map (Fig. 3A), all 150 patients were classified into three subgroups. Among them, subgroup1 and subgroup2 were categorized into one group - Subtype1, while subgroup3 was denoted by Subtype2. In addition, the Kaplan-Meier curves showed that either OS or RFS period of Subtype2 was significantly longer than that of Subtype1 with the Log-rank *P* values being 0.029 and 0.005, respectively (Fig. 3B).

**Figure 4:**
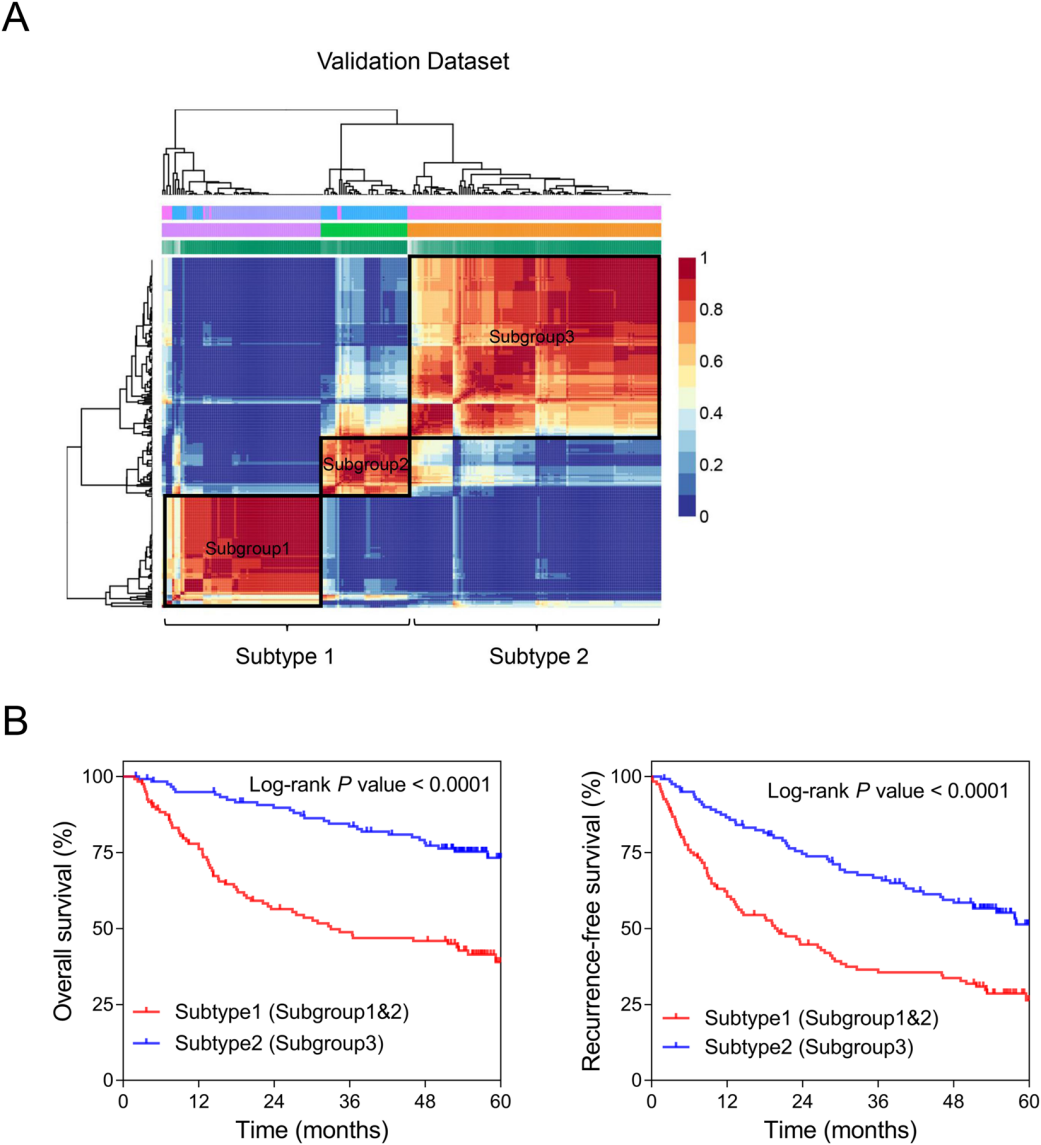
Validation of the constructed molecular subtypes in GSE14520. (**A**) The NMF clustering consensus map of the validation set (GSE14520) based on the expression of the same gene set as used in TCGA cohort. The molecular subtyping method was consistent with that of the training set. (**B**) The Kaplan-Meier analyses for the OS (*Left*) and RFS (*Right*) of Subtype1 and Subtype2 patients in the validation dataset. *P* values were generated from Log-rank tests.

To validate the robustness of the constructed molecular subtyping system, same clustering approach based on the 185-gene set was used in GSE14520. Similarly, subgroup1 and subgroup2 were classified into molecular Subtype1 and subgroup3 was considered as Subtype2 (Fig. 4A). Moreover, the survival curves showed patients in Subtype2 had remarkably better prognosis than Subtype1 participants (Log-rank *P* < 0.001) (Fig. 4B). These evidence showed that the molecular subtype was not only unique in genetic features but also closely related to the clinical outcome in Asian hepatectomy patients.

**Figure 5:**
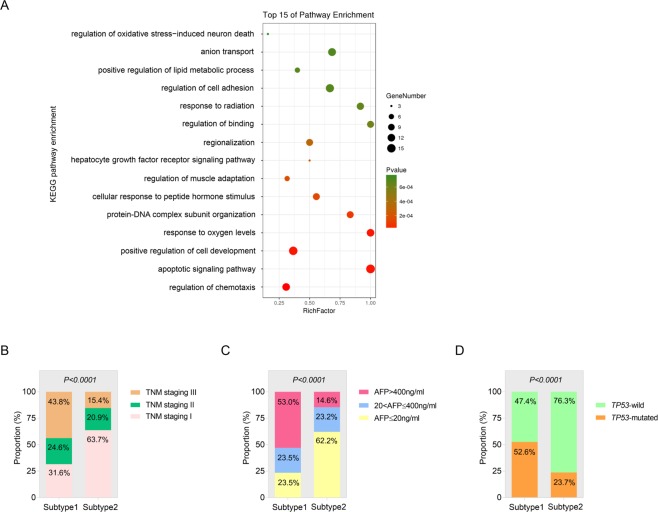
Evaluation of the biological functions and clinical significance of the constructed molecular signature of Asian HCC. (**A**) Bubble plot of the enriched KEGG pathways. (**B**–**D**) Associations between the molecular subtype and TNM stage (**B**) preoperative serum AFP level (**C**) and *TP53* mutation status (**D**).

To explore potential biological functions of the 185 HCC-characteristic genes, Kyoto Encyclopedia of Genes and Genomes (KEGG) pathways were enriched. The most enriched pathways were as follows: regulation of chemotaxis, apoptotic signaling pathway, positive regulation of cell development and some microenvironmental conditions that cellular development needs (oxygen levels, hormone stimulus, etc.), cell proliferation-associated pathways such as protein-DNA complex subunit organization, hepatocyte growth factor receptor, etc. Besides, pathways related with cell migration were enriched as well like regulation of binding and cell adhesion (Fig. 5A).

Furthermore, the relations between the molecular subtype and clinical characteristics were also examined using the clinical information and mutation profiles of the TCGA cohorts. It was shown that the proportions of patients in late TNM stage (TNM II, III) (Fig. 5B) and with higher level of preoperative alpha-fetoprotein (AFP) (Fig. 5C) in Subtype1 molecular group was significantly larger than those in Subtype2 group (*P* < 0.0001). With respect to *TP53* mutation status, the ratio of *TP53*-mutated patients in Subtype1 group was notably higher than that in Subtype2 group (*P* < 0.0001) (Fig. 5D). Taken together, our results supported the findings presented above that the molecular Subtype2 patients were more likely to exhibit favorable outcome while Subtype1 were associated with poorer prognosis.

**Figure 6:**
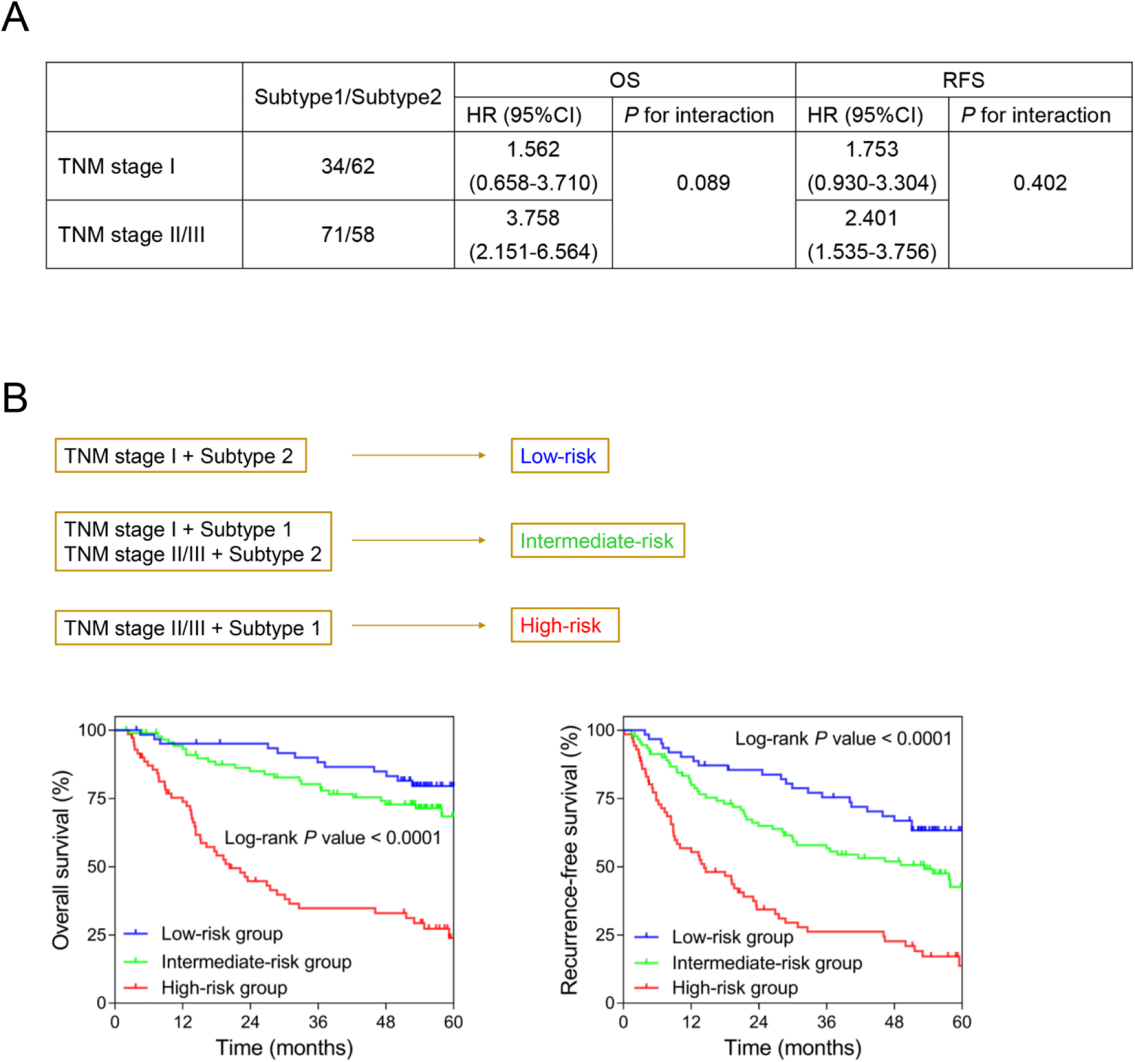
Combination of the molecular subtyping system and TNM stages for risk stratification. (**A**) The results of stratified survival analysis for OS and RFS of different TNM-stage HCC and interaction between the molecular subtype and TNM stage using GSE14520. (**B**) Kaplan-Meier curves for OS (*Left*) and RFS (*Right*) of the low-risk, intermediate-risk and high-risk patients. The survival rates by time of three risk groups were compared using Log-rank tests for *P* values.

To refine prognostic prediction, we integrated TNM stage and molecular subtype for risk classification based on the clinical information of the validation set (GSE14520). Firstly, the interaction assessed between TNM stage and molecular subtype was non-significant, either for OS or RFS (*P* > 0.05), with all HRs (Subtype1/Subtype2) from stratified survival analysis being more than 1, indicating our molecular subtyping system is robust in patients of different tumor stages (Fig. 6A). After augmenting TNM stage, patients were classified into low-risk (Subtype2 + TNM stage I), intermediate-risk (Subtype2 + TNM stage II/III, Subtype1 + TNM stage I) and high-risk (Subtype1 + TNM stage II/III) groups. Kaplan-Meier curves showed remarkable differences (Log-rank *P* < 0.05) of the OS and RFS between three risk groups providing evidence that integration of the molecular subtype and TNM stage would improve postoperative risk stratification of Asian HCC patients receiving liver resection (Fig. 6B).

## Discussion

In this study, we identified a molecular subtyping system of Asian HCC which could predict postoperative survival and recurrence. Unlike previous studies using only few genes^9,10^, our molecular signature was comprised of a large panel of 185 HCC-characteristic prognostic genes screened by a comprehensive analysis of the whole gene set profiles and follow-up information of each participant. This method made the identified molecular subtype more representative of the intrinsic heterogeneity of the cancer on the basis of its clinical significance. It is common that molecular features are opposite of clinical or pathological markers^11^. To challenge this opposition, we integrated the clinical staging system and the molecular system into stratified postsurgical survival analysis which proved highly effective in risk stratification.

It is noteworthy that a few ICC and CHC samples were closely genetically related to some HCC samples from PCA analysis (Fig. 2A). This finding supported the published results in a latest study by Dr. Wang that in CHC samples, HCC and ICC components shared the common cell origin^12^. On account of the genetic similarity between HCC and the other two types of PLC, the high specificity of the molecular classification of HCC was in demand. In this study, we used Venn selection to identify the DEGs merely in HCC versus non-tumor tissue and significantly associated with patients’ OS and RFS simultaneously as HCC-characteristic genes. These specific genes were used for the further molecular subtype establishment which improved the specificity of our molecular signature to a great extent.

Although the exact biological functions of most genes in the identified HCC-characteristic gene set are still unclear, the possible functions of the whole gene set have been predicted here. The KEGG pathway enrichment revealed that these genes correlated with the regulation of chemotaxis and cell apoptosis and development. Nonetheless, these findings indicated that the HCC-characteristic genes were worth further studying as the important candidates in the molecular mechanism of HCC progression and potential therapeutic targets in HCC treatment.

In conclusion, this study developed a molecular subtyping system for predicting the prognosis of Asian HCC patients after liver resection via NMF clustering. This molecular signature was robust and effective in the validation analysis using another independent dataset and gave new implications for the construction of novel prognostic assessment approaches rather than limited to a few genes. Our findings would promote the tailored therapy of HCC patients as well though more external validation is needed.

## Materials and Methods

### Data preparation and pre-processing

Raw data of four datasets, GSE15765, GSE5851, GSE28691 and GSE32879, were downloaded from Gene Expression Omnibus (GEO) database (https://www.ncbi.nlm.nih.gov/geo/). GSE15765, GSE5851 and GSE28691 were all performed by the same chip platform, GPL571 (Affymetrix Human Genome U133A 2.0 Array). While GSE32879 was conducted by GPL6244, also known as Affymetrix Human Gene 1.0 ST Array [transcript (gene) version]. These four cohorts were comprised of different subtypes of liver cancer and non-tumor liver tissue samples. After background adjustment and normalization within each dataset and between datasets^13^, the comparable data of 70 HCC samples, 29 ICC samples, 14 CHC samples, 75 MLC samples and 7 non-tumor samples were acquired (Fig. 1). The training dataset was obtained from The Cancer Genome Atlas (TCGA) database (https://cancergenome.nih.gov/). And a total of 150 Asian patients who received liver resection for HCC with complete clinical information were enrolled. Another independent dataset from GEO, GSE14520, was employed as the validation dataset. In this dataset, 242 eligible HCC patients were recruited. GSE14520 was performed using Affymetrix Human Genome U133A and U133 Plus 2.0 Arrays. The normalization method for the raw data of GSE14520 was consistent with that for the datasets (by GPL571) described above.

### Screening of the HCC-characteristic genes

Differentially expressed genes (DEGs) between three histological types of primary liver cancer (HCC, ICC and CHC) and non-tumor samples were compared by a multiple *t*-test. *P* values were corrected for FDR and FDR <0.05 was considered statistically significant. The calculation was performed in R software (version 3.4.0). Those genes which were significantly expressed between HCC and non-tumor samples while non-significantly expressed between the other two types and non-tumor tissue were retrieved for the following Cox survival analysis. Univariate Cox analysis was conducted to screen the prognostic genes significantly associated with the OS and RFS of hepatectomy patients. Venn selection (http://bioinfogp.cnb.csic.es/tools/venny/) was used to identify the HCC-characteristic prognostic genes.

### Non-negative matrix factorization consensus clustering

NMF clustering was carried out using the R package ‘NMF’ (http://cran.r-project.org/package=NMF) in R software^14^. The number of runs was set at 40 and the number of clusters *k* was chosen as 3 in this present study when the consensus map and the cophenetic and silhouette coefficients were simultaneously observed relatively satisfactory^8^. The patient subgroups were determined by the NMF clusters based on the gene expression profiles. The three subgroups were classified into two molecular subtypes with two subgroups who have similar gene set profiles combined as a single molecular subtype. The survival curves were plotted using Kaplan-Meier analysis. The OS and RFS of two risk groups were compared by Log-rank test.

### Pathway enrichment analysis

The biological functions of the identified characteristic genes were predicted based on the KEGG pathways. The KEGG pathway enrichment was conducted via a free online tool for bioinformatic analysis (http://www.omicshare.com/tools). The *P* values and RichFactors for enriched pathways were calculated for evaluation.

### Statistical analysis

Based on the gene expression value, PCA among different types of liver cancer and non-tumor tissue was carried out using R software (version 3.4.0). Comparisons between the distribution of the transformed categorical variables were performed through Chi-square test or Mann–Whitney *U* test, as appropriate. The Kaplan-Meier analysis were performed in GraphPad Prism version 7.0. The stratified Cox survival analysis was conducted in patients in different TNM stages. The interaction between the identified molecular subtype and TNM staging was tested using R. All the statistical calculation was carried out using SPSS version 23.0 (IBM Corporation, Armonk, NY, USA), unless otherwise indicated. *P* value (2-tailed) less than 0.05 was defined as a significance threshold.

## Supplementary information

Table S1

## Data Availability

The datasets used in this manuscript could be accessible through https://www.ncbi.nlm.nih.gov/geo/ and https://portal.gdc.cancer.gov/.
