# Supplementary material for: Identification of a molecular subtyping system associated with the prognosis of Asian hepatocellular carcinoma patients receiving liver resection
Source: Sci Rep. 2019 May 8;9:7073. doi: 10.1038/s41598-019-43548-1 (PMC6506502; doi:10.1038/s41598-019-43548-1)
Supplement: Supplementary file 1 — Table S1 [file 41598_2019_43548_MOESM1_ESM.docx]

**Identification of a molecular subtyping system associated with the prognosis of Asian hepatocellular carcinoma patients receiving liver resection**

**Running title**: *prognostic molecular subtypes in Asian HCC after hepatectomy*

Xiaohua Ma^1, *^, Jingxian Gu^1, *^, Kun Wang^2, *^, Xing Zhang^1^, Juan Bai^3^, Jingyao Zhang^1^, Chang Liu^1^, Qiang Qiu^2^, Kai Qu^1^

**Author Affiliations:**

^1^ Department of Hepatobiliary Surgery, The First Affiliated Hospital of Xi’an Jiaotong University, Xi’an 710061, Shaanxi, China.

^2^ Center for Ecological and Environmental Sciences, Key Laboratory for Space Bioscience and Biotechnology, Northwestern Polytechnical University, Xi'an, 710072, China.

^3^ Department of Immunology, Shaanxi University of Chinese Medicine, Xianyang Shaanxi 712046, People's Republic of China.

^*^ Authors (X.M., J.G. and K.W.) shared co-first authorship.

**# Correspondence should be addressed to:**

**Kai Qu** Ph.D., M.D.

Address: Department of Hepatobiliary Surgery, The First Affiliated Hospital of Xi’an Jiaotong University, Xi’an 710061, Shaanxi, China. Tel & Fax: +86-029-8532 3900.

Email: qukai@xjtu.edu.cn.

Table S1 List of 185 differentially expressed genes1

| **probeset ID** | **gene symbol** | **gene title** | **LogFC^a^** | **FDR^b^** |
| --- | --- | --- | --- | --- |
| 215600_x_at | FBXW12 | F-box and WD repeat domain containing 12 | 2.282 | 4.21E-46 |
| 204162_at | NDC80 | NDC80 kinetochore complex component | 2.201 | 8.9E-34 |
| 205046_at | CENPE | centromere protein E, 312kDa | 1.869 | 7.65E-33 |
| 216527_at | HCG18 | HLA complex group 18 (non-protein coding) | 1.547 | 1.23E-32 |
| 204521_at | FAM216A | family with sequence similarity 216, member A | 1.686 | 6.24E-30 |
| 217887_s_at | EPS15 | epidermal growth factor receptor pathway substrate 15 | 0.780 | 1.31E-29 |
| 200957_s_at | SSRP1 | structure specific recognition protein 1 | 2.100 | 1.21E-24 |
| 219123_at | ZNF232 | zinc finger protein 232 | 1.995 | 1.3E-24 |
| 220454_s_at | SEMA6A | sema domain, transmembrane domain (TM), and cytoplasmic domain, (semaphorin) 6A | 0.566 | 7.72E-24 |
| 212075_s_at | CSNK2A1 | casein kinase 2, alpha 1 polypeptide | 0.814 | 4.71E-23 |
| 215341_at | DNAH6 | dynein, axonemal, heavy chain 6 | 1.499 | 1.44E-22 |
| 211333_s_at | FASLG | Fas ligand (TNF superfamily, member 6) | 1.280 | 2.11E-22 |
| 208285_at | OR7A5 | olfactory receptor, family 7, subfamily A, member 5 | 1.345 | 2.34E-22 |
| 205352_at | SERPINI1 | serpin peptidase inhibitor, clade I (neuroserpin), member 1 | 1.749 | 2.03E-21 |
| 210722_at | PNLIPRP1 | pancreatic lipase-related protein 1 | 1.225 | 1.06E-17 |
| 206197_at | NME5 | NME/NM23 family member 5 | 1.318 | 2.64E-17 |
| 209032_s_at | CADM1 | cell adhesion molecule 1 | 1.288 | 5.09E-17 |
| 219888_at | SPAG4 | sperm associated antigen 4 | 1.528 | 1.31E-16 |
| 219308_s_at | AK5 | adenylate kinase 5 | 1.189 | 3.45E-16 |
| 204776_at | THBS4 | thrombospondin 4 | 1.448 | 6.16E-16 |
| 205235_s_at | KIF20B | kinesin family member 20B | 1.971 | 1.5E-15 |
| 215518_at | STXBP5L | syntaxin binding protein 5-like | 1.133 | 2.7E-14 |
| 208240_s_at | FGF1 | fibroblast growth factor 1 (acidic) | 1.343 | 2.75E-14 |
| 220581_at | CCDC170 | coiled-coil domain containing 170 | 1.408 | 9.98E-14 |
| 220812_s_at | HHLA2 | HERV-H LTR-associating 2 | 1.127 | 1.38E-13 |
| 219426_at | AGO3 | argonaute RISC catalytic component 3 | 0.831 | 3.65E-13 |
| 220643_s_at | FAIM | Fas apoptotic inhibitory molecule | 1.524 | 1E-12 |
| 215368_at | NEB | nebulin | 1.650 | 2.86E-12 |
| 220716_at | ERCC8 | excision repair cross-complementation group 8 | 1.712 | 3.15E-12 |
| 219425_at | SULT4A1 | sulfotransferase family 4A, member 1 | 1.522 | 8.49E-12 |
| 206612_at | CACNG1 | calcium channel, voltage-dependent, gamma subunit 1 | 1.552 | 1.17E-11 |
| 203832_at | SNRPF | small nuclear ribonucleoprotein polypeptide F | 2.017 | 1.22E-11 |
| 202806_at | TOM1 | drebrin 1 | 0.720 | 2.89E-11 |
| 220651_s_at | MCM10 | minichromosome maintenance complex component 10 | 1.412 | 3.63E-11 |
| 221493_at | TSPYL1 | TSPY-like 1 | 2.382 | 4.66E-11 |
| 220150_s_at | FAM184A | family with sequence similarity 184, member A | 1.449 | 5.09E-11 |
| 220813_at | CYSLTR2 | cysteinyl leukotriene receptor 2 | 1.472 | 1.93E-10 |
| 213254_at | TNRC6B | trinucleotide repeat containing 6B | 0.813 | 2.09E-10 |
| 206051_at | ELAVL4 | ELAV like neuron-specific RNA binding protein 4 | 1.198 | 4.25E-10 |
| 205260_s_at | ACYP1 | acylphosphatase 1, erythrocyte (common) type | 1.205 | 5.33E-10 |
| 207772_s_at | PRMT8 | protein arginine methyltransferase 8 | 1.245 | 1.37E-09 |
| 220194_at | NSUN7 | NOP2/Sun domain family, member 7 | 0.876 | 2.15E-09 |
| 218484_at | NDUFA4L2 | NADH dehydrogenase (ubiquinone) 1 alpha subcomplex, 4-like 2 | 1.358 | 2.27E-09 |
| 204580_at | MMP12 | matrix metallopeptidase 12 (macrophage elastase) | 1.848 | 4.15E-09 |
| 214759_at | WTAP | Wilms tumor 1 associated protein | 1.279 | 0.000000019 |
| 205488_at | GZMA | granzyme A (granzyme 1, cytotoxic T-lymphocyte-associated serine esterase 3) | 1.470 | 2.17E-08 |
| 220741_s_at | PPA2 | pyrophosphatase (inorganic) 2 | 1.892 | 2.35E-08 |
| 206749_at | CD1B | CD1b molecule | 1.254 | 2.65E-08 |
| 208747_s_at | C1S | complement component 1, s subcomponent | 0.934 | 3.14E-08 |
| 220647_s_at | COA4 | cytochrome c oxidase assembly factor 4 homolog (S. cerevisiae) | 1.433 | 3.94E-08 |
| 220255_at | FANCE | Fanconi anemia, complementation group E | 1.179 | 7.63E-08 |
| 213998_s_at | DDX17 | DEAD (Asp-Glu-Ala-Asp) box helicase 17 | 0.644 | 0.000000174 |
| 205242_at | CXCL13 | chemokine (C-X-C motif) ligand 13 | 1.652 | 0.000000195 |
| 222108_at | AMIGO2 | adhesion molecule with Ig-like domain 2 | 1.360 | 0.000000455 |
| 221658_s_at | IL21R | interleukin 21 receptor | 0.856 | 0.00000053 |
| 210164_at | GZMB | granzyme B (granzyme 2, cytotoxic T-lymphocyte-associated serine esterase 1) | 0.797 | 0.00000109 |
| 213697_at | HIPK3 | homeodomain interacting protein kinase 3 | 0.584 | 0.00000121 |
| 214023_x_at | TUBB2B | tubulin, beta 2B class IIb | 0.671 | 0.00000155 |
| 205299_s_at | BTN2A2 | butyrophilin, subfamily 2, member A2 | 0.702 | 0.00000304 |
| 216825_s_at | MPL | MPL proto-oncogene, thrombopoietin receptor | 0.892 | 0.00000331 |
| 218804_at | ANO1 | anoctamin 1, calcium activated chloride channel | 0.770 | 0.00000347 |
| 218145_at | TRIB3 | tribbles pseudokinase 3 | 1.485 | 0.00000372 |
| 213859_x_at | SMARCA5 | SWI/SNF related, matrix associated, actin dependent regulator of chromatin, subfamily a, member 5 | 0.841 | 0.00000406 |
| 211578_s_at | RPS6KB1 | ribosomal protein S6 kinase, 70kDa, polypeptide 1 | 0.820 | 0.00000425 |
| 200948_at | MLF2 | myeloid leukemia factor 2 | 0.853 | 0.00000524 |
| 217928_s_at | PPP6R3 | protein phosphatase 6, regulatory subunit 3 | 0.819 | 0.00000547 |
| 202567_at | SNRPD3 | small nuclear ribonucleoprotein D3 polypeptide 18kDa | 1.084 | 0.00000826 |
| 205204_at | NMB | neuromedin B | 1.514 | 0.0000131 |
| 211122_s_at | CXCL11 | chemokine (C-X-C motif) ligand 11 | 1.452 | 0.0000213 |
| 218678_at | NES | nestin | 1.321 | 0.0000215 |
| 208113_x_at | PABPC3 | poly(A) binding protein, cytoplasmic 3 | 1.655 | 0.000022 |
| 206765_at | KCNJ2 | potassium inwardly-rectifying channel, subfamily J, member 2 | 1.321 | 0.0000243 |
| 219138_at | RPL14 | ribosomal protein L14 | 1.600 | 0.0000294 |
| 220688_s_at | MRTO4 | mRNA turnover 4 homolog (S. cerevisiae) | 1.179 | 0.0000317 |
| 204533_at | CXCL10 | chemokine (C-X-C motif) ligand 10 | 1.816 | 0.0000334 |
| 213357_at | GTF2H5 | general transcription factor IIH, polypeptide 5 | 1.564 | 0.0000344 |
| 218308_at | TACC3 | transforming, acidic coiled-coil containing protein 3 | 1.313 | 0.0000398 |
| 214499_s_at | BCLAF1 | BCL2-associated transcription factor 1 | 0.767 | 0.000061 |
| 220431_at | TMPRSS11E | transmembrane protease, serine 11E | 1.182 | 0.0000662 |
| 210923_at | SLC1A7 | solute carrier family 1 (glutamate transporter), member 7 | 1.296 | 0.0000759 |
| 216454_at | TRMT1 | tRNA methyltransferase 1 homolog (S. cerevisiae) | 1.114 | 0.0000764 |
| 213728_at | LAMP1 | lysosomal-associated membrane protein 1 | 0.875 | 0.0000777 |
| 213727_x_at | MPPE1 | metallophosphoesterase 1 | 1.216 | 0.000099 |
| 203022_at | RNASEH2A | ribonuclease H2, subunit A | 1.280 | 0.000119647 |
| 217280_x_at | GABRA5 | gamma-aminobutyric acid (GABA) A receptor, alpha 5 | 0.698 | 0.000128191 |
| 206310_at | SPINK2 | serine peptidase inhibitor, Kazal type 2 (acrosin-trypsin inhibitor) | 1.300 | 0.000179592 |
| 202107_s_at | MCM2 | minichromosome maintenance complex component 2 | 1.300 | 0.0002132 |
| 222206_s_at | NCLN | nicalin | 0.619 | 0.000214149 |
| 206417_at | CNGA1 | cyclic nucleotide gated channel alpha 1 | 1.643 | 0.000220264 |
| 204617_s_at | ACD | adrenocortical dysplasia homolog (mouse) | 1.244 | 0.00022123 |
| 208510_s_at | PPARG | peroxisome proliferator-activated receptor gamma | 1.276 | 0.000301901 |
| 205287_s_at | TFAP2C | transcription factor AP-2 gamma (activating enhancer binding protein 2 gamma) | 1.202 | 0.000302893 |
| 214795_at | ZMYND8 | zinc finger, MYND-type containing 8 | 0.529 | 0.000323635 |
| 209615_s_at | PAK1 | p21 protein (Cdc42/Rac)-activated kinase 1 | 0.699 | 0.000355876 |
| 201892_s_at | IMPDH2 | IMP (inosine 5'-monophosphate) dehydrogenase 2 | 1.189 | 0.000388331 |
| 212368_at | ZNF292 | zinc finger protein 292 | 0.768 | 0.000399682 |
| 206245_s_at | IVNS1ABP | influenza virus NS1A binding protein | 0.840 | 0.000737281 |
| 218284_at | SMAD3 | SMAD family member 3 | 0.683 | 0.000812785 |
| 220891_at | TRMT44 | tRNA methyltransferase 44 homolog (S. cerevisiae) | 0.606 | 0.000824418 |
| 201075_s_at | SMARCC1 | SWI/SNF related, matrix associated, actin dependent regulator of chromatin, subfamily c, member 1 | 0.672 | 0.000831119 |
| 214057_at | MCL1 | myeloid cell leukemia 1 | 0.634 | 0.000983851 |
| 207381_at | ALOX12B | arachidonate 12-lipoxygenase, 12R type | 0.757 | 0.001143938 |
| 222039_at | KIF18B | kinesin family member 18B | 1.318 | 0.001168198 |
| 212151_at | PBX1 | pre-B-cell leukemia homeobox 1 | 0.580 | 0.001216479 |
| 220139_at | DNMT3L | DNA (cytosine-5-)-methyltransferase 3-like | 1.168 | 0.001517729 |
| 207263_x_at | VEZT | vezatin, adherens junctions transmembrane protein | 0.685 | 0.001577597 |
| 212298_at | NRP1 | neuropilin 1 | 0.786 | 0.001643729 |
| 218741_at | CENPM | centromere protein M | 1.213 | 0.001662865 |
| 211756_at | PTHLH | parathyroid hormone-like hormone | 0.854 | 0.001905199 |
| 221350_at | HOXC8 | homeobox C8 | 1.358 | 0.001937992 |
| 222383_s_at | ALOXE3 | arachidonate lipoxygenase 3 | 1.254 | 0.001999471 |
| 204088_at | P2RX4 | purinergic receptor P2X, ligand-gated ion channel, 4 | 1.336 | 0.002072028 |
| 214623_at | FBXW4P1 | F-box and WD repeat domain containing 4 pseudogene 1 | 0.780 | 0.002187034 |
| 221699_s_at | DDX50 | DEAD (Asp-Glu-Ala-Asp) box polypeptide 50 | 1.187 | 0.002485437 |
| 218995_s_at | EDN1 | endothelin 1 | 1.239 | 0.00253622 |
| 219280_at | BRWD1 | bromodomain and WD repeat domain containing 1 | 0.688 | 0.002575377 |
| 201568_at | UQCRQ | ubiquinol-cytochrome c reductase, complex III subunit VII, 9.5kDa | 1.243 | 0.002652946 |
| 214101_s_at | NPEPPS | aminopeptidase puromycin sensitive | 0.875 | 0.002781002 |
| 200779_at | ATF4 | activating transcription factor 4 | 1.468 | 0.002800472 |
| 217968_at | TSSC1 | tumor suppressing subtransferable candidate 1 | 1.243 | 0.002836934 |
| 221370_at | ZNF717 | zinc finger protein 717 | 0.421 | 0.00302741 |
| 41397_at | ZNF821 | zinc finger protein 821 | 1.203 | 0.003081372 |
| 208803_s_at | SRP72 | signal recognition particle 72kDa | 1.145 | 0.003551417 |
| 201795_at | LBR | lamin B receptor | 1.081 | 0.003660217 |
| 213030_s_at | PLXNA2 | plexin A2 | 0.761 | 0.004207175 |
| 214513_s_at | CREB1 | cAMP responsive element binding protein 1 | 0.817 | 0.004620369 |
| 213252_at | SH3PXD2A | SH3 and PX domains 2A | 0.459 | 0.00463989 |
| 207051_at | SLC17A4 | solute carrier family 17, member 4 | 0.842 | 0.004991785 |
| 204086_at | PRAME | preferentially expressed antigen in melanoma | 1.263 | 0.005260304 |
| 210505_at | ADH7 | alcohol dehydrogenase 7 (class IV), mu or sigma polypeptide | 1.168 | 0.005747426 |
| 204351_at | S100P | S100 calcium binding protein P | 1.359 | 0.005884141 |
| 221696_s_at | STYK1 | serine/threonine/tyrosine kinase 1 | 0.826 | 0.006093496 |
| 200713_s_at | MAPRE1 | microtubule-associated protein, RP/EB family, member 1 | 0.834 | 0.006118929 |
| 200804_at | TMBIM6 | transmembrane BAX inhibitor motif containing 6 | 0.836 | 0.006321237 |
| 214899_at | ZNF780B | zinc finger protein 780B | 0.446 | 0.006533169 |
| 212852_s_at | TROVE2 | TROVE domain family, member 2 | 0.497 | 0.006591493 |
| 219357_at | GTPBP1 | GTP binding protein 1 | 0.574 | 0.006859419 |
| 202090_s_at | UQCR11 | ubiquinol-cytochrome c reductase, complex III subunit XI | 1.479 | 0.006876608 |
| 206894_at | APOA4 | apolipoprotein A-IV | 1.518 | 0.007302317 |
| 213137_s_at | PTPN2 | protein tyrosine phosphatase, non-receptor type 2 | 0.529 | 0.008179257 |
| 221705_s_at | SIKE1 | suppressor of IKBKE 1 | 0.509 | 0.008304507 |
| 203161_s_at | RNF8 | ring finger protein 8, E3 ubiquitin protein ligase | 1.355 | 0.008443462 |
| 218622_at | NUP37 | nucleoporin 37kDa | 1.492 | 0.009083769 |
| 208570_at | WNT1 | wingless-type MMTV integration site family, member 1 | 0.580 | 0.009694802 |
| 218295_s_at | NUP50 | nucleoporin 50kDa | 1.550 | 0.009770974 |
| 221871_s_at | TFG | TRK-fused gene | 1.308 | 0.010960062 |
| 220927_s_at | HPSE2 | heparanase 2 (inactive) | 1.109 | 0.011043981 |
| 209143_s_at | CLNS1A | chloride channel, nucleotide-sensitive, 1A | 1.427 | 0.011234572 |
| 213834_at | IQSEC3 | IQ motif and Sec7 domain 3 | 0.521 | 0.011293059 |
| 204230_s_at | SLC17A7 | solute carrier family 17 (vesicular glutamate transporter), member 7 | 1.246 | 0.01161423 |
| 219031_s_at | NIP7 | NIP7, nucleolar pre-rRNA processing protein | 0.805 | 0.012915198 |
| 207556_s_at | DGKZ | diacylglycerol kinase, zeta | 1.245 | 0.014114063 |
| 207786_at | CYP2R1 | cytochrome P450, family 2, subfamily R, polypeptide 1 | 0.617 | 0.014228949 |
| 206674_at | FLT3 | fms-related tyrosine kinase 3 | 1.120 | 0.01542609 |
| 221509_at | DENR | density-regulated protein | 1.556 | 0.016905314 |
| 216845_x_at | KMT2D | lysine (K)-specific methyltransferase 2D | 0.469 | 0.017402266 |
| 207115_x_at | MBTD1 | mbt domain containing 1 | 0.579 | 0.018226345 |
| 204717_s_at | SLC29A2 | solute carrier family 29 (equilibrative nucleoside transporter), member 2 | 0.684 | 0.019054282 |
| 206225_at | ZNF507 | zinc finger protein 507 | 0.567 | 0.01978483 |
| 206607_at | CBL | Cbl proto-oncogene, E3 ubiquitin protein ligase | 0.596 | 0.02081608 |
| 222158_s_at | DESI2 | desumoylating isopeptidase 2 | 1.322 | 0.021366733 |
| 216555_at | PRR14L | proline rich 14-like | 0.664 | 0.023089982 |
| 221819_at | RAB35 | RAB35, member RAS oncogene family | 0.805 | 0.024174306 |
| 203540_at | GFAP | glial fibrillary acidic protein | 0.720 | 0.026690755 |
| 204324_s_at | GOLIM4 | golgi integral membrane protein 4 | 0.605 | 0.027202411 |
| 213620_s_at | ICAM2 | intercellular adhesion molecule 2 | 1.149 | 0.029176117 |
| 201200_at | CREG1 | cellular repressor of E1A-stimulated genes 1 | 1.162 | 0.029861277 |
| 216967_at | GAP43 | growth associated protein 43 | 0.813 | 0.030700989 |
| 221935_s_at | EOGT | EGF domain-specific O-linked N-acetylglucosamine (GlcNAc) transferase | 1.213 | 0.030982678 |
| 204885_s_at | MSLN | mesothelin | 0.726 | 0.031442153 |
| 210814_at | TRPC3 | transient receptor potential cation channel, subfamily C, member 3 | 1.094 | 0.032319673 |
| 206603_at | SLC2A4 | solute carrier family 2 (facilitated glucose transporter), member 4 | 0.631 | 0.032711193 |
| 202150_s_at | NEDD9 | neural precursor cell expressed, developmentally down-regulated 9 | 1.508 | 0.032748601 |
| 203103_s_at | PRPF19 | pre-mRNA processing factor 19 | 0.954 | 0.035311725 |
| 211594_s_at | MRPL9 | mitochondrial ribosomal protein L9 | 1.308 | 0.03587023 |
| 213970_at | RABL3 | RAB, member of RAS oncogene family-like 3 | 0.822 | 0.036378281 |
| 202223_at | STT3A | STT3A, subunit of the oligosaccharyltransferase complex (catalytic) | 0.793 | 0.039226042 |
| 35666_at | SEMA3F | sema domain, immunoglobulin domain (Ig), short basic domain, secreted, (semaphorin) 3F | 0.749 | 0.04031369 |
| 218470_at | YARS2 | tyrosyl-tRNA synthetase 2, mitochondrial | 1.269 | 0.04153168 |
| 204589_at | NUAK1 | NUAK family, SNF1-like kinase, 1 | 1.359 | 0.042107884 |
| 205863_at | S100A12 | S100 calcium binding protein A12 | 1.118 | 0.04224091 |
| 205656_at | PCDH17 | protocadherin 17 | 1.147 | 0.045912002 |
| 213608_s_at | SRRD | SRR1 domain containing | 1.153 | 0.046301459 |
| 218140_x_at | SRPRB | signal recognition particle receptor, B subunit | 1.270 | 0.04877954 |
| 202622_s_at | ATXN2 | ataxin 2 | 0.808 | 0.049632884 |

^a^: logFC was the logarithm Fold Change as HCC tissue being compared with non-tumor tissue. +/- represented up-/down- regulated expression level in HCC.

^b^: False discovery rate.
